# Inhibition of thrombin generation in human plasma by phospholipid transfer protein

**DOI:** 10.1186/s12959-015-0054-0

**Published:** 2015-07-16

**Authors:** Hiroshi Deguchi, Gertrud Wolfbauer, Marian C. Cheung, Yajnavalka Banerjee, Darlene J. Elias, José A. Fernández, John J. Albers, John H. Griffin

**Affiliations:** Department of Molecular and Experimental Medicine, The Scripps Research Institute, MEM180, 10550 North Torrey Pines Rd., La Jolla, CA 92037 USA; Division of Metabolism, Endocrinology, and Nutrition, Northwest Lipid Metabolism and Diabetes Research Laboratories, Department of Medicine, University of Washington, Seattle, WA 98109 USA; Current Address: Department of Biochemistry, College of Medicine and Health Sciences, SQ University, Muscat, Oman

**Keywords:** Phospholipid transfer protein, Factor XII, Venous thromboembolism, Thrombin generation

## Abstract

**Background:**

Plasma phospholipid transfer protein (PLTP) transfers lipids between donors and acceptors (*e.g.*, from HDL to VLDL) and modulates lipoprotein composition, size, and levels. No study has reported an assessment of the effects of PLTP on blood clotting reactions, such as reflected in thrombin generation assays, or on the association of venous thrombosis (VTE) risk with PLTP activity.

**Methods:**

The *in vitro* effects of PLTP on blood coagulation reactions and the correlations between plasma PLTP activity levels and VTE were studied.

**Results:**

Recombinant (r) PLTP concentration-dependently inhibited plasma thrombin generation and factor XII-dependent kallikrein generation when sulfatide was used to stimulate factor XII autoactivation in plasma. However, rPLTP did not inhibit thrombin generation in plasma induced by factor XIa or tissue factor, implicating an effect of PLTP on contact activation reactions. In purified systems, rPLTP inhibited factor XII autoactivation stimulated by sulfatide in the presence of VLDL. In surface plasmon resonance studies, purified factor XII bound to immobilized rPLTP, implying that rPLTP inhibits factor XII-dependent contact activation by binding factor XII in the presence of lipoproteins. Analysis of plasmas from 40 male patients with unprovoked VTE and 40 matched controls indicated that low PLTP lipid transfer activity (≤25th percentile) was associated with an increased risk of VTE after adjustment for body mass index, plasma lipids, and two known thrombophilic genetic risk factors.

**Conclusion:**

These data imply that PLTP may be an antithrombotic plasma protein by inhibiting generation of prothrombotic factor XIIa in the presence of VLDL. This newly discovered anticoagulant activity of PLTP merits further clinical and biochemical studies.

**Electronic supplementary material:**

The online version of this article (doi:10.1186/s12959-015-0054-0) contains supplementary material, which is available to authorized users.

## Background

The blood coagulation system is triggered by either the intrinsic or extrinsic pathway and involves sequential enzymatic activations of serine protease zymogens enhanced by non-enzymatic cofactors, factors Va and VIIIa, resulting in generation of thrombin [1, 2]. Thrombin generation is central to the regulation of hemostasis and thrombosis and to the pathogenesis of cardiovascular disease and venous thrombosis. The intrinsic pathway is triggered by contact activation. Recent studies using mouse thrombosis models suggest that the intrinsic pathway is essential for pathological thrombus formation in both the arterial [3–7] and venous systems [7]. The “contact activation” of plasma involves 3 zymogens, factor XII, factor XI and prekallikrein, plus a non-enzymatic cofactor, high molecular weight kininogen, that participate in a set of interrelated proteolytic activation reactions [4, 8–15]. During the initial stages of contact activation, factor XII and prekallikrein participate in reciprocal proteolysis in which factor XIIa activates prekallikrein to kallikrein, which in turn converts factor XII into factor XIIa. Activation of factor XII can be enhanced by autoactivation of zymogen factor XII by the active form α-factor XIIa. These reactions are accelerated by negatively charged surfaces (*e.g.*, kaolin, sulfatides, and dextran sulfate).

Plasma lipids and lipoproteins can influence both procoagulant and anticoagulant reactions [16, 17]. Furthermore, dyslipidemia and dyslipoproteinemia are associated with hypercoagulability and venous thromboembolism (VTE) [16, 17]. The lipid transfer protein, cholesteryl ester transfer protein (CETP), which carries and transfers lipids to modulate plasma lipoprotein levels, can exert procoagulant activity by enhancing prothrombinase activity in purified systems [18]. Furthermore, plasma CETP mass level was correlated with relative hypercoagulability of plasma independent of high density lipoprotein (HDL) levels. Although molecular mechanisms for CETP procoagulant activity remain unclear, we hypothesized that other lipid transfer proteins can also affect blood coagulation.

Plasma phospholipid transfer protein (PLTP), a homolog of CETP, circulates in plasma and facilitates the transfer of phospholipids and cholesterol among lipoproteins [19–27]. It can mediate the conversion of HDL into larger and smaller particles [28, 29] and generate pre-β HDL in the process [30]. PLTP also transfers phospholipids from very low density lipoprotein (VLDL) to HDL by PLTP shuttling from HDL to VLDL particles [31]. In clinical studies, high PLTP activity was reported to be associated with the increased risk of coronary artery disease [32, 33], notably in statin-treated patients [34]. In contrast, low PLTP activity was reported to be associated with peripheral atherosclerosis [35], suggesting that the relationship of plasma levels of PLTP activity to cardiovascular risk is controversial [32–38]. No study has reported an assessment of the effects of PLTP on blood clotting reactions, such as reflected in thrombin generation assays, or on the association of VTE risk with PLTP activity.

Here we report that PLTP can inhibit sulfatide-induced contact activation of thrombin generation, that factor XII binds directly to PLTP, and that low plasma PLTP activity levels may be associated with VTE risk.

## Methods

### Materials

Fatty acid-free bovine serum albumin (BSA) was purchased from Calbiochem (San Diego, CA). Human factor XIa and corn trypsin inhibitor were from Hematologic Technologies Inc. (Essex Junction, VT). Human factor XII, prekallikrein and kallikrein were from Enzyme Research Laboratories (South Bend, IN). Human α-factor XIIa was from Aniara (Mason, OH). VLDL was purchased from Intracel (Frederic, MD). Sulfatide was obtained from Matreya (Pleasant Gap, PA). Kaolin was from Fisher Scientific Inc. (Pittsburgh, PA) and dextran sulfate was from GE Healthcare (Parsippany NJ). Innovin was from DADE (BioMerioeux). Chromogenic substrate S2302 and fluorogenic substrate I-1140 were obtained from Chromogenix (Franklin, OH) and Bachem Bioscience Inc. (King of Prussia, PA), respectively. Normal human pooled plasma was prepared using blood obtained from 20 adult healthy donors (10 males and 10 females) by routine venipuncture from the Scripps General Clinical Research Center’s (GCRC) blood donation program after an overnight fast. Blood was mixed with 0.129 M sodium citrate at 1:9 ratios. Plasma was prepared by centrifugation at 2,000 × g for 20 min at room temperature from each donor and pooled. The pooled normal human plasma was stored at −80 °C. Blood from VTE patients was collected in the GCRC at least 3 months after VTE diagnosis and after a 12 h fast.

### Recombinant (r) PLTP

Recombinant wild-type PLTP was made and characterized as full length molecules as reported [29, 39]. Briefly, rPLTP was isolated by Ni^2+^-nitrilotriacetic acid resin column chromatography from serum-free conditioned culture medium collected from baby hamster kidney cells transfected with a His-tagged human rPLTP cDNA using methotrexate as selection agent. The isolated rPLTP fractions were assayed for phospholipid transfer activity and evaluated for purity by SDS-PAGE. The concentration of rPLTP was determined by the absorbance at 280 nm. rPLTP was stabilized by adding phosphatidylcholine vesicles in Tris-buffered saline (TBS) containing 0.05 M Tris, 0.15 M NaCl, pH 7.4. The same buffer containing the same concentration of phosphatidylcholine vesicles without rPLTP was used as control for activity assays.

### Preparation of sulfatide vesicles

Sulfatide vesicles were prepared by sonication and stored up to two days. Briefly, 1 mg/ml bovine sulfatide in TBS was sonicated 5 times for 30 s with 1 min intervals using the ultrasonic processor XL (Heat System, Inc., Farmingdale, NY) under the flow of N_2_.

### Thrombin generation assay in plasma

Plasma thrombin generation assays were performed as described with some modifications [40]. Pooled normal human plasma (30 μl) was incubated with various rPLTP concentrations for 15 min at 37 °C. Then, tissue factor (Innovin, final 4 pM) or sulfatide vesicles containing 30 mM CaCl_2_ and fluorogenic thrombin substrate solution (I-1140) was added to the plasma mixture (total 110 μl) to initiate coagulation activation. In factor XIa (0.13 nM, final) -induced thrombin generation assays, corn trypsin inhibitor (50 μg/ml, final) was also pre-incubated with plasma. Thrombin generation was followed continuously using SPECTRAmax GEMINI XS fluorometer (Molecular Devices, Sunnyvale, CA) with excitation and emission wavelengths set at 360 and 460 nm, respectively. The first derivative of fluorescence versus time was used to produce thrombin generation curves.

### Kallikrein generation assay in plasma

The generation of plasma kallikrein in the presence of sulfatide was determined as described with some modifications [8]. Briefly, plasma (1:100, final dilution) was incubated with PLTP for 15 min at room temperature, followed by the addition of sulfatide (0.5 μM, final) and then incubated at various times. Kallikrein amidolytic activity was measured by hydrolysis of the kallikrein amidolytic substrate, S2302 (0.4 mM, final). Soy bean trypsin inhibitor (500 μg/ml, final), which inhibits kallikrein activity, inhibited S2302 hydrolysis in this assay system by approximately 95 %, whereas corn trypsin inhibitor, which specifically inhibits factor XIIa, inhibited S2302 hydrolysis by 5 % (data not shown). These showed that the S2302 hydrolysis by contact phase activation in this plasma assay system mainly reflects kallikrein generation rather factor XIIa generation due to the different reactivities of kallikrein and factor XIIa with the substrate [13].

### Factor XII activation in purified protein system

For factor XII autoactivation assays, factor XII (0.1 μM, final) was pre-incubated with rPLTP in the presence or absence of VLDL (25 μg protein/mL, final) for 15 min at room temperature, and then sulfatide vesicles (0.5 μM, final) were added to start autoactivation of factor XII. S2302 (0.4 mM, final) was added at various time points and the amidolytic activity of factor XIIa was measured [8, 9].

Activation of factor XII by kallikrein was measured as described [10]. The reaction mixture containing factor XII (0.1 μM, final) with or without rPLTP (5 μg/mL, final) in the presence or absence of VLDL (25 μg protein/mL, final) was pre-incubated for 15 min at 37 °C, followed by the addition of kallikrein (0.4 nM, final) and incubated for optimal time. The reaction was stopped by adding soybean trypsin inhibitor (500 μg/ml final) to block kallikrein. The generated factor XIIa activity was measured as amidolytic activity for S2302 (0.4 mM, final).

### Prekallikrein activation by factor XIIa

Activation of prekallikrein by factor XIIa was measured as described [12]. The reaction mixture containing prekallikrein (0.25 μM, final) with or without rPLTP (5 μg/mL, final) in the assay buffer was pre-incubated in the presence or absence of VLDL (25 μg protein/mL, final) for 15 min at 37 °C, followed by the addition of factor XIIa (0.1 nM, final) and incubated for optimal times. The kallikrein activity generated was measured as amidolytic activity for S2302 (0.4 mM, final).

### Factor XII binding to PLTP

Binding was assessed by surface plasmon resonance (SPR) analysis using a BIAcore 3000 biosensor system. An anti-His tag monoclonal antibody was covalently immobilized on the carboxymethylated dextran (CM5) sensor chip (BIAcore) using amine coupling chemistry according to the manufacturer’s instructions. A nonreactive mouse IgG was used as a control for nonspecific binding. rPLTP with a C-terminal His-tag (100 μg/ml) was diluted in 50 mM Hepes, 150 mM NaCl, and 5 mM CaCl_2_ (pH 7.4) and injected at a flow rate of 10 μL/min with 10 min contact time generating a response. Then, each concentration of factor XII was injected in this buffer for 1.5 min at a flow rate of 5 μL/min. After each Factor XII sensorgram was obtained, the His tag-antibody surface was regenerated with 10 mM glycine/HCl, pH 2.5, and a new injection of His-tag PLTP was used to regenerate the surface. Any influence of mass transport effects was discounted from the results of binding and dissociation at different flow rates. Data analysis was performed by using BIAevaluation software 3.0 (BIAcore). The association and dissociation phases of all ‘sensorgrams’ were fitted globally. Rate constants for association (k_a_) and dissociation (k_d_) were obtained by globally fitting the data from five to six injections of factor XII (0–750 nM) by using the BIAevaluation software version 3.2, using the simple Langmuir binding model.

### VTE study group

The Scripps Venous Thrombosis Registry is an ongoing case–control study of risk factors for VTE as described [41]. Inclusion criteria for this study included age at thrombosis < 55 years old, >3 months since diagnosis of acute thrombosis, a life expectancy of at least 3 years and no lipid lowering medications or cancer. Age matched (±2 years) healthy male controls were recruited through the GCRC blood donation program at Scripps. The protocol was approved by the Institutional Review Board of Scripps Clinic and subjects provided written informed consent. Forty of 49 VTE patients (82 %) presented with idiopathic VTE, defined as events that did not occur within 90 days after surgery, trauma, or major immobilization. In this study, male idiopathic VTE patients (*n* = 40) and controls (*N* = 40) were analyzed for PLTP activity and mass (Additional file 1: Table S1).

### Clinical analytes

Serum lipid profile data were obtained from the routine clinical lab using standard techniques.

Lipoprotein subclass particle concentrations were measured by Nuclear Magnetic Resonance Spectroscopy (NMR) at LipoScience, Inc (Raleigh,NC) [41]. PLTP activity (total plasma activity) and mass were determined as described [23, 26].

### Statistical analysis

Data for VTE patients and controls was compared for median values using the Mann–Whitney test using Prism™ 4.0 software (Graph Pad Software Inc., San Diego, CA). Odds ratios for VTE were determined with a logistic regression model using Minitab software. The difference was considered significant when p was < 0.05.

## Results

### Effects of rPLTP on sulfatide-induced plasma thrombin generation

**Fig. 1:**
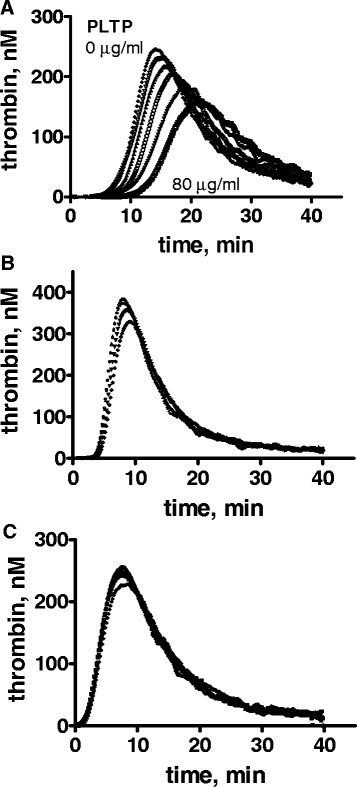
Inhibition of thrombin generation by rPLTP. Normal pooled human plasma was mixed with various concentrations of rPLTP, and thrombin generation was initiated by adding the following procoagulant stimuli with 30 mM CaCl_2_: **a** sulfatides (20 μM); **b** factor XIa (0.13 nM final) plus corn trypsin inhibitor; or **c** tissue factor. Concentrations of rPLTP used with different procoagulant stimuli were: **a** 0, 5, 10, 20, 40 and 80 μg/ml corresponding to the lines from left to right; **b** 0, 5 (top two lines which overlap each other), 10 and 40 μg/ml corresponding to the lines from top to bottom; and **c** 0, 5 and 10 (three lines which overlap each other), and 40 μg/ml of rPLTP (bottom line)

When added to plasma in increasing amounts, rPLTP markedly reduced the total amount of thrombin generation and prolonged the lag time for thrombin generation in plasma activated by sulfatides (Fig. 1a). In contrast, rPLTP had no effect or only a very slight effect on factor XIa-induced or tissue factor-induced thrombin generation in plasma (Figs. 1b and c). This suggests that rPLTP significantly inhibits the factor XII-dependent contact activation reactions but not the intrinsic or extrinsic coagulation pathways activated by factor XIa or tissue factor.

### Effect of increasing sulfatide concentration on inhibition of thrombin generation in plasma by rPLTP

**Fig. 2:**
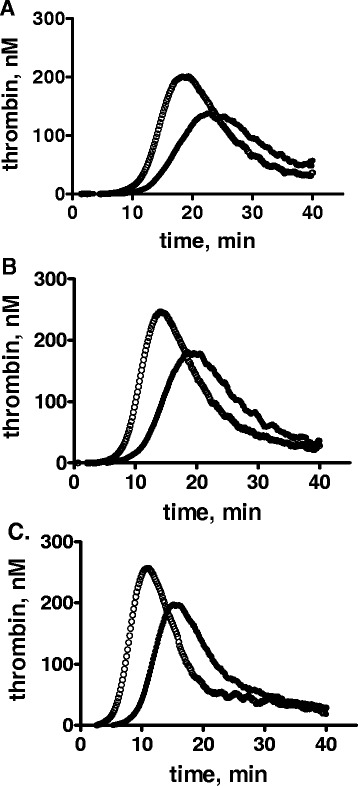
Inhibition of thrombin generation by rPLTP at various concentrations of sulfatide. Normal pooled human plasma was mixed with rPLTP or control buffer and thrombin generation was initiated by adding procoagulant sulfatide with 30 mM CaCl_2_ at the following levels: **a** 2 μM; **b** 20 μM; or **c** 100 μM. The rPLTP concentration was 0 (○) or 40 μg/ml (●)

To study if the effect of rPLTP is due to interactions of rPLTP with sulfatide, the effect of rPLTP was tested under various concentrations of sulfatide (2 μM, 20 μM and 100 μM). The ability of rPLTP to inhibit thrombin generation was not neutralized or significantly altered by increasing the amounts of sulfatide (*e.g.*, from 2 μM to 100 μM) (Fig. 2), indicating that the major effect of PLTP was independent of sulfatide concentration. Besides sulfatides, other negatively charged surfaces (*e.g.*, kaolin, dextran sulfate, *etc.*) also stimulate contact activation. When contact activation was stimulated by kaolin or dextran sulfate, rPLTP did not inhibit thrombin generation in plasma (Additional file 1: Figure S1).

### Effect of rPLTP on sulfatide-induced kallikrein generation in plasma

**Fig. 3:**
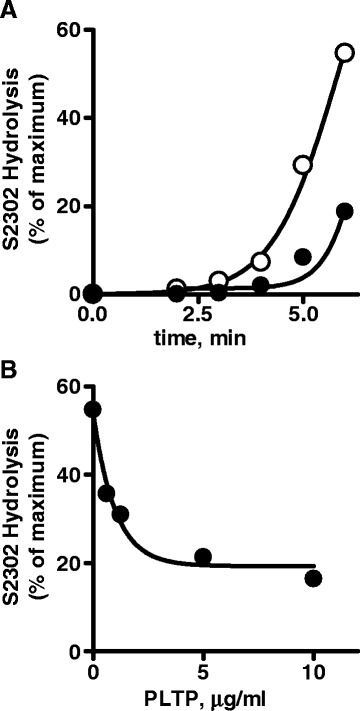
Inhibition of sulfatide-induced kallikrein generation by rPLTP in plasma. **a** The time course of inhibition of sulfatide-dependent kallikrein generation by PLTP is shown as kallikrein amidolytic activity (S2302). The rPLTP concentration was 0 (○) or 5 μg/ml (●). **b** The rPLTP dose response for inhibition of sulfatide-dependent kallikrein generation by rPLTP in plasma is shown based on kallikrein activity determined at 6 min after kallikrein generation was initiated by adding (0.5 μM) sulfatides

To study the effect of rPLTP on contact activation reactions, the sulfatide-induced activation of prekallikrein/factor XII in plasma was evaluated at various time points. In the assay system employed, contact activation was monitored by kallikrein generation. In controls, rPLTP (0–20 μg/ml) did not directly affect the kallikrein amidolytic activity towards the chromogenic substrate S2302 (data not shown). rPLTP delayed generation of plasma kallikrein activity by sulfatides in plasma (Fig. 3a) and did so in a concentration-dependent fashion (IC_50_ = 0.69 μg/ml) (Fig. 3b). These data show that rPLTP inhibits contact phase activation in the coagulation system.

### Effect of rPLTP on contact phase activation

**Fig. 4:**
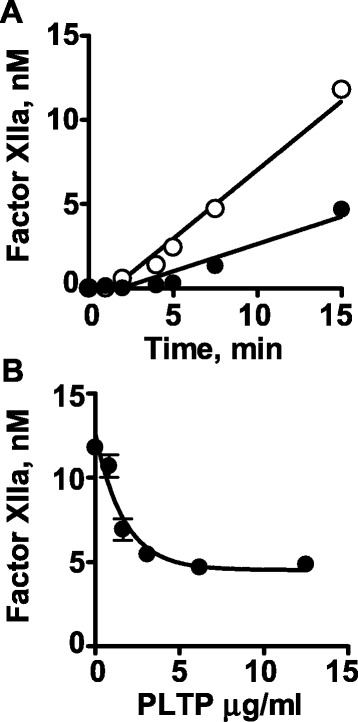
Inhibition of sulfatide-stimulated FXII autoactivation by rPLTP in the presence of VLDL. **a** The time course of inhibition of sulfatide-stimulated factor XII autoactivation by rPLTP was determined when factor XII (0.1 μM, final) was pre-incubated with rPLTP (5 μg/mL) in the presence of VLDL (25 μg/ml protein, final) for 15 min at room temperature, and sulfatide vesicles (final 0.5 μg/ml) were added to start autoactivation of factor XII. Then, S2302 was added at various time points and the amidolytic activity was measured at different time points. The rPLTP concentration was 0 (○) or 5 μg/ml (●). **b** The dose–response for rPLTP inhibition of sulfatide-stimulated factor XII autoactivation by rPLTP was determined as in panel (**a**) and data for factor XIIa activity at 15 min are shown

Contact phase activation in the coagulation system is enhanced by factor XII autoactivation and by the reciprocal activations of factor XII by kallikrein and of prekallikrein by factor XIIa. When the effect of rPLTP on contact activation reactions was studied in assays containing only purified protein components, rPLTP did not affect factor XII auto-activation stimulated by sulfatide, factor XII activation by kallikrein, or prekallikrein activation by factor XIIa (Additional file 1: Figure S2, A, B, and C, respectively). However, when VLDL was added into the purified reaction component mixture, rPLTP inhibited factor XII autoactivation (Fig. 4a) in a concentration-dependent fashion (IC_50_ = 1.2 μg/ml) (Fig. 4b). Neither Factor XII activation by kallikrein nor prekallikrein activation by factor XIIa was affected by rPLTP in the absence or presence of VLDL (Additional file 1: Figure S3 A and B). The amidolytic activity of FXIIa or kallikrein towards S2302 was not inhibited by PLTP even in the absence or presence of VLDL (data not shown).

### rPLTP binding to factor XII

**Fig. 5:**
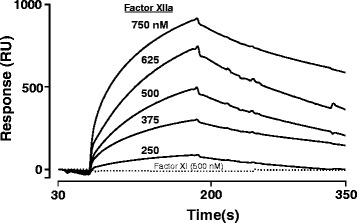
Binding of Factor XII to rPLTP using SPR. SPR was used to monitor binding of factor XII (250–750 nM) to immobilized rPLTP. Sensorgrams represent the binding of factor XII (from top to bottom at 750, 625, 500, 375, 250 nM, as indicated. In controls, purified Factor XI (500 nM) (dotted line) did not exhibit any binding to immobilized rPLTP

SPR was used to study the binding of factor XII to rPLTP. Factor XII bound to immobilized rPLTP whereas no binding of factor XI (up to 500 nM) to rPLTP was detected by SPR (Fig. 5). Based on the data analysis, rate constants k_on_ and k_off_ for binding of factor XII to rPLTP and its apparent Kd were 0.31 × 10^5^ (1/Ms), 60 × 10^−4^ (1/s) and 190 nM, respectively.

### Association of venous thrombotic disease with PLTP activity and mass

Because rPLTP can inhibit thrombin generation in plasma, we postulated that low plasma levels of PLTP may be linked to VTE. In a pilot study of subjects from the Scripps VTE Registry, we assessed whether low levels of plasma PLTP are associated with VTE risk. There was no significant difference in the median values of PLTP activity or PLTP mass levels in 40 adult male VTE patients compared to matched controls (*p* = 0.57 and 0.59, respectively) (data not shown). When the quartile-based odds ratio (OR) for the association of VTE with PLTP activity was calculated by comparing quartile 1 (lowest PLTP) with quartiles 2–4 (Table 1), a low level of PLTP activity was not statistically significantly associated with increased VTE risk although the OR was 2.2 (95%CI: 0.78-5.9) (Table 1, model I). Some plasma parameters which may be associated with both PLTP level and VTE risk, namely BMI and HDL-C (Additional file 1: Table S1) (23), and some other lipids possibly linked to procoagulant activities which may be altered by PLTP were used for adjustment of the OR. When adjusted for BMI and HDL-C, the OR for low PLTP activity was 2.7 (95%CI: 0.91-7.8, *p* = 0.07) (Table 1, model II), and further adjustment for LDL-C and triglyceride (TG) improved the OR to 5.2 (95%CI: 1.5-19, p = 0.01) (Table 1, model IV). Particularly significant was the finding that the low PLTP activity level was highly significantly associated with the risk of VTE with an OR = 12 (95%CI: 2.4-56, *p* = 0.002) after further adjustment for the known VTE risk factors, FV Leiden and prothrombin nt20210A (Table 1, model V). When VLDL particle concentrations measured by NMR was used for the correction instead of TG, the ORs were similar to the values with triglyceride (OR = 5.3 (95%CI: 1.5-19) for model IV and OR = 11(95%CI: 2.3-52) for model V, respectively). The high PLTP levels (>75 percentile) was not protective against VTE (OR = 1.4 (95%CI: 0.55-3.8), *p* = 0.53) even after adjustment described above (OR = 0.75 (95%CI: 0.23-2.5) for model IV and OR = 0.65 (95%CI: 0.17-2.5) for model V, respectively). PLTP mass was not associated with the risk of VTE based on quartile-based OR analysis in any of the models summarized in Table 1.

**Table 1:** Quartile-based Odds Ratio (OR) for association of low PLTP activity (≤25th percentile)

	PLTP activity		PLTP mass	
Model and adjustments	OR (95%CI)	p	OR (95%CI)	p
I. No adjustment	2.2 (0.78-5.9)	0.15	1.3 (0.46-3.8)	0.59
II. BMI + HDL	2.7 (0.91- 7.8)	0.07	0.53 (0.14-2.1)	0.55
III. II + FV Leiden + prothrombin nt20210A	3.5 (1.1-12)	0.04	0.49 (0.1-1.8)	0.37
IV II + LDL-C + TG	5.2 (1.5-19)	0.01	0.84 (0.23-3.0)	0.79
V. III+ LDL-C + TG	12 (2.4-56)	0.002	0.62 (0.15-2.6)	0.52

The odds ratios (**OR**) for unprovoked VTE with low levels (≤25th percentile) of PLTP activity and PLTP mass are shown. Models were adjusted by variables as indicated, including BMI, HDL-C, LDL-C and TG which were used as continuous values and factor V Leiden and prothrombin nt20210A

## Discussion

In this study we showed that a recombinant plasma protein, rPLTP, can inhibit sulfatide-induced thrombin generation and contact activation in plasma, implying that it may function as an anticoagulant factor where contact activation is involved. This activity of rPLTP was not apparent when thrombin generation in plasma was triggered by factor XIa or tissue factor, implying a direct effect of rPLTP on contact activation reactions. Factor XII is the key enzyme for contact activation [1–7] and, indeed, we found that factor XII binds directly to rPLTP, thereby providing a starting point for mechanistic studies for the actions of rPLTP.

Interestingly, rPLTP did not inhibit contact activation in plasma stimulated by two other negatively charged activators of the contact system, dextran sulfate and kaolin, further implying that the anticoagulant actions of rPLTP were not due to a nonspecific blocking of negative charges. These findings might be supported by reports that mechanisms responsible for surface activation triggered by different negatively charged molecules (*e.g.*, sulfatide and kaolin) differ from each other as regards the molecular interaction with the contact factors [11, 14]. However, the details for the mechanistic relations of PLTP to surface materials for contact activation of coagulation including other naturally occurring negatively charged surfaces effect (*e.g.*, misfolded proteins, and polyphosphates) need to be evaluated in future studies.

The requirement of VLDL, which also could provide surface for contact pathway of coagulation system [42], for the inhibition of sulfatide-induced autoactivation of factor XII by rPLTP in purified protein reaction mixtures may reflect direct or indirect effects of VLDL on factor XII and/or rPLTP. Recent work shows that a significant fraction of PLTP is bound to VLDL in plasma [39], so the ability of rPLTP to inhibit sulfatide-induced contact activation in plasma or factor XII autoactivation likely reflects actions of a PLTP●VLDL complex in plasma. Extensive and detailed mechanistic studies using rPLTP, factor XII and α-factor XIIa plus VLDL and many proteins that associate with VLDL [43] would be needed for studies to clarify details and elucidate the structural basis for the effects of these molecules on contact activation or factor XII autoactivation. Further, other lipoproteins (*i.e.*, HDL and LDL), which were not tested here, could also possibly contribute to the anticoagulant activity of PLTP.

Binding studies using purified proteins show direct interactions between factor XII and PLTP. For this protein-protein interaction, the apparent Kd value of 190 nM is below the plasma level of factor XII which is 300 nM [44] but above the plasma level of PLTP at 25 nM [19]. Perhaps reflecting this modest value of apparent Kd, PLTP alone does not inhibit sulfatide-induced factor XII autoactivation in purified reaction systems. However, when VLDL was added to the purified system, PLTP does inhibit Factor XII autoactivation, showing additional plasma components are needed. Thus, because VLDL particles carry many associated proteins in addition to PLTP [43], we speculate that the whole VLDL particles or one or more proteins in the VLDL interactome enhances affinity of PLTP for factor XIIa which at very low levels is responsible for triggering factor XII autoactivation. Additionally, factor XII conformational changes induced by sulfatide or zinc ions [14] might influence the affinity of factor XIIa for PLTP. However, mechanisms for inhibition of factor XII autoactivation by PLTP in plasma are not completely clear and they need further clarifications including the potential influences of VLDL, sulfatide, or zinc ions on factor XIIa-PLTP binding interactions.

Factor XII is not required for normal hemostasis in man or various animals. However, studies of thrombosis injury models in mice genetically deficient in factor XII or XI suggest that factor XII-dependent thrombin generation via the intrinsic coagulation pathway can significantly contribute *in vivo* to fibrin formation and thrombosis including a pulmonary embolism model [3–7]. This concept plus the ability of PLTP to inhibit thrombin generation in plasma led us to assay PLTP activity and mass in plasmas of a young adult male VTE cohort that we have previously described [23, 41]. The initial analysis of our data without any adjustments for variables failed to show any association of plasma PLTP activity and mass levels with VTE risk. However, a very significant association between low PLTP activity and VTE became apparent after making adjustments for various lipoprotein levels (Table 1, models IV and V) or by analyzing separately the subgroup of normolipidemic patients (Additional file 1: Figure S4). *I*t appears that levels of plasma lipoproteins, which are indeed related to PLTP-mediated phospholipid transfer activity [23], can mask the association of PLTP activity with VTE risk. Overall, our finding here is consistent with the concept that active PLTP molecules, putatively in VLDL●PLTP complexes [43], contribute to the multiple, highly varied antithrombotic activities of plasma. One notes that major limitations of this pilot VTE study include the low number of subjects, the age of subjects under 55 years old, and the absence of female cohorts.

Factor XII-dependent contact activation not only might contribute *in vivo* to fibrin formation and excessive thrombosis including pulmonary embolism model [3–7] but also might contribute to inflammation and pathologies via bradykinin formation and complement activation [5, 6, 45]. Thus, inhibition of contact activation mediated by PLTP might inhibit not only thrombosis but also inflammatory processes stimulated or supported by contact activation. In this regard, it is interesting that PLTP was shown to exert anti-inflammatory activities [46, 47], although no information relating PLTP’s anti-inflammatory actions to any pro-inflammatory actions due to contact activation have been described.

## Conclusions

We found that PLTP is an inhibitor of sulfatide-initiated factor XII-dependent thrombin generation by inhibiting factor XII autoactivation and that VLDL appears to contribute to this activity of PLTP. After adjustments for two thrombophilic genetic risk factors and for lipoprotein and lipid parameters known to be affected by plasma PLTP activity, low plasma PLTP activity was very significantly associated with increased risk of VTE in a small pilot study of young adult normolipidemic males. Both the functionally significant interactions between PLTP and factor XII and the relationship between PLTP activity and VTE merit further detailed investigations.

## Additional file

Additional file 1: Table S1. Scripps Registry VTE Study population. **Figure S1.** PLTP Inhibition of thrombin or kallikrein generation stimulated by kaolin or dextran sulfate in plasma. Normal pooled human plasma (30 μl) was mixed with rPLTP (0 (○) or 40 μg/ml (●), respectively), and thrombin generation was initiated by adding kaolin (0.25 mg/ml) (A) or dextran sulfate (25 μg/ml) (B) with 30 mM CaCl2. **Figure S2.** Absence of inhibition by rPLTP of sulfatide-stimulated FXII autoactivation or of factor XII activation by kallikrein or of prekallikrein activation by factor XIIa in the absence of VLDL in purified systems. (A) Time course of sulfatide-stimulated factor XII autoactivation. (B) Time course of factor XII activation by kallikrein. (C) Time course of prekallikrein activation by factor XIIa. The rPLTP concentration was 0 (○) or 5 μg/ml (●). **Figure S3.** Absence of inhibition by rPLTP of sulfatide-stimulated factor XII activation by kallikrein or of prekallikrein activation by factor XIIa in the presence of VLDL in purified system. (A) Time course of factor XII activation by kallikrein. (B) Time course of prekallikrein activation by factor XIIa. The rPLTP concentration was 0 (○) or 5 μg/ml (●). **Figure S4.** The distribution of PLTP activity in VTE and control subgroups when patients and controls were stratified as hyperlipidemic (+) and normolipidemic (-) based on LDL-C levels. The hyperlipidemia group (+) was defined as subjects who had LDL–C or TG levels that were greater than the 75 percentile of controls.
