# Supplementary material for: Inhibition of thrombin generation in human plasma by phospholipid transfer protein
Source: Thromb J. 2015 Jul 16;13:24. doi: 10.1186/s12959-015-0054-0 (PMC4504036; doi:10.1186/s12959-015-0054-0)
Supplement: Additional file 1: Table S1. — Scripps Registry VTE Study population. Figure S1. PLTP Inhibition of thrombin or kallikrein generation stimulated by kaolin or dextran sulfate in plasma. Normal pooled human plasma (30 μl) was mixed with rPLTP (0 (○) or 40 μg/ml (●), respectively), and thrombin generation was initiated by adding kaolin (0.25 mg/ml) (A) or dextran sulfate (25 μg/ml) (B) with 30 mM CaCl2. Figure S2. Absence of inhibition by rPLTP of sulfatide-stimulated FXII autoactivation or of factor XII activation by kallikrein or of prekallikrein activation by factor XIIa in the absence of VLDL in purified systems. (A) Time course of sulfatide-stimulated factor XII autoactivation. (B) Time course of factor XII activation by kallikrein. (C) Time course of prekallikrein activation by factor XIIa. The rPLTP concentration was 0 (○) or 5 μg/ml (●). Figure S3. Absence of inhibition by rPLTP of sulfatide-stimulated factor XII activation by kallikrein or of prekallikrein activation by factor XIIa in the presence of VLDL in purified system. (A) Time course of factor XII activation by kallikrein. (B) Time course of prekallikrein activation by factor XIIa. The rPLTP concentration was 0 (○) or 5 μg/ml (●). Figure S4. The distribution of PLTP activity in VTE and control subgroups when patients and controls were stratified as hyperlipidemic (+) and normolipidemic (-) based on LDL-C levels. The hyperlipidemia group (+) was defined as subjects who had LDL–C or TG levels that were greater than the 75 percentile of controls. [file 12959_2015_54_MOESM1_ESM.docx]

**Online Supplementary Material**

**H. Deguchi et al.
Inhibition of thrombin generation in human plasma by phospholipid transfer protein.**

**Supplemental Table**

**Table. S1 Scripps Registry VTE Study population**

|  | ***Control*** | ***VTE*** | **p value** |
| --- | --- | --- | --- |
| **Variables** | **N=40** | **N=40** |  |
| **Age, yr (SD)** | 45.9 (8.4) | 46.3 (8.9) | 0.72 |
| **Male** | 40 (100) | 40 (100) |  |
| **Races, %** |  |  |  |
| **Caucasian** | 90.0 | 92.5 | 0.69 |
|  |  |  |  |
| **Body-mass Index, kg m^-2^  (SD)** | 27.5 (4.4) | 30.3 (5.2) | **0.01** |
|  |  |  |  |
| **Risk factors, number of subjects (%)** |  |  |  |
| **Factor V Leiden** | 2 (5.0) | 11 (27.5) | **0.006** |
| **Prothrombin 20210A** | 1 (2.5) | 7 (14.3) | **0.03** |
|  |  |  |  |
| **Idiopathic VTE (%)** |  | 40 (100) |  |
|  |  |  |  |
| **Clinical Lipids, mean (SD)** |  |  |  |
| **total cholesterol, mg/dl** | 204 (42) | 218 (45) | 0.12 |
| **Triglyceride, mg/dl** | 147 (89) | 163 (104) | 0.58 |
| **HDL-C, mg/dl** | 53.3 (15.9) | 46.9 (18.5) | **0.01** |
| **LDL-C, mg/dl** | 120 (33) | 135 (42) | 0.11 |

To evaluate the association of VTE with categorized factors; ethnic group and genetic polymorphisms, χ^2^ analysis was used. Mann-Whitney was where performed for lipid parameters and student t-test was performed for age and BMI.

**Supplemental Figures**

**Figure S1. PLTP Inhibition of thrombin or kallikrein generation stimulated by kaolin or dextran sulfate in plasma.** Normal pooled human plasma (30 μl) was mixed with rPLTP (0 (○) or 40 µg/ml (●), respectively), and thrombin generation was initiated by adding kaolin (0.25 mg/ml) **(A)** or dextran sulfate (25 μg/ml) **(B)** with 30 mM CaCl_2_.

**Figure S2.** **Absence of inhibition by rPLTP of sulfatide-stimulated FXII autoactivation or of factor XII activation by kallikrein or of prekallikrein activation by factor XIIa in the absence of VLDL in purified systems. (A)** Time course of sulfatide-stimulated factor XII autoactivation. (**B)** Time course of factor XII activation by kallikrein. (**C)** Time course of prekallikrein activation by factor XIIa. The rPLTP concentration was 0 (○) or 5 µg/ml (●).

**Figure S3.** **Absence of inhibition by rPLTP of sulfatide-stimulated factor XII activation by kallikrein or of prekallikrein activation by factor XIIa in the presence of VLDL in purified system. (A)** Time course of factor XII activation by kallikrein.

(**B)** Time course of prekallikrein activation by factor XIIa. The rPLTP concentration was 0 (○) or 5 µg/ml (●).

**Figure S4.** **The distribution of PLTP activity in VTE and control subgroups when patients and controls were stratified as hyperlipidemic (+) and normolipidemic (-) based on LDL-C levels.** The hyperlipidemia group (**+**) was defined as subjects who had LDL–C or TG levels that were greater than the 75 percentile of controls.
